# The impact of the reaction to diagnosis on sibling relationship: a study on parents and adult siblings of people with disabilities

**DOI:** 10.3389/fpsyg.2025.1551953

**Published:** 2025-05-07

**Authors:** Flavia Lecciso, Chiara Martis, Giuseppe Antonioli, Annalisa Levante

**Affiliations:** ^1^Department of Human and Social Sciences, University of Salento, Via di Valesio, Lecce, Italy; ^2^Lab of Applied Psychology, Department of Human and Social Sciences, University of Salento, Via di Valesio, Lecce, Italy

**Keywords:** reaction to diagnosis, sibling, parents, disability, sibling relationship, resolution

## Abstract

**Objective:**

Building on Bowlby's attachment theory and Marvin and Pianta's framework, the current study investigated the role of the resolution of the diagnosis as a potential protective factor in shaping the quality of affective sibling relationships. The study examined whether the typically developing (TD) siblings' resolution of the diagnosis of their brother/sister with a disability would predict the quality of their affective relationship in terms of closeness, conflict, jealousy, self-marginalization, and worry (HP1). The potential predictive role of parental resolution on typically developing sibling resolution has been investigated (HP2). In addition, we explored whether being an older *vs*. younger typically developing sibling would impact the resolution of the diagnosis and the quality of the affective sibling relationship.

**Methods:**

A total of 365 parent–sibling dyads [parents: *M*_*age*_(*SD*) = 51.2 (6.95) years, age range = 25–64 years; mothers = 78.4%; TD siblings: *M*_*age*_(*SD*) = 23.2 (3.60) years, age range = 18–39 years; females = 53.7%] from families of individuals with neurodevelopmental disorders or physical disabilities filled out the e-survey (CE n° 92,949/2,023).

**Results:**

The results supported both hypotheses, highlighting the potential protective role of typically developing siblings' resolution of the diagnosis in fostering high-quality sibling relationships in terms of high closeness and low conflict, jealousy, self-marginalization, and worry. In addition, parental resolution of the diagnosis emerged as a potential predictor of typically developing siblings' resolution, supporting the intergenerational transmission of internal working models. Regarding the research question (RQ), younger typically developing siblings reported higher resolution scores than their older counterparts, suggesting that typically developing sibling birth order may shape the reaction to the diagnosis process.

**Conclusion:**

The results underscored the importance of systemic interventions that devote attention not only to parental resolution but also to that of typically developing siblings. Future research should deepen the role played by socio-demographic factors and long-term outcomes on typically developing siblings' mental health and caregiving duties.

## 1 Introduction

Sibling relationships are often the longest-lasting and most significant ones in individuals' lives (Connidis and Campbell, 1995; Goetting, 1986). Generally, in typically developing (TD) conditions, siblings are caregivers, friends, and confidants (Lobato, 1990). In the context of disability, the sibling relationship requires special attention. In this vein, evidence (Orsmond et al., 2009; Ross and Cuskelly, 2006) reported that typically developing siblings (TD siblings) in each developmental stage (from childhood to adulthood) assumed adult-like caregiving roles, providing to their brother/sister with disabilities not only care but also emotional support in managing household duties.

In the context of disabilities, there is inconsistent evidence regarding the impact of disabilities on sibling relationships. During the lifespan, closeness, sharing, love, support, respect, and personal growth, as well as conflict and rivalry, were the main qualitative sibling relationship characteristics extracted from interviews (Bhattashali et al., 2018; Noonan et al., 2018; Paul et al., 2022; Stock et al., 2016; Tyerman et al., 2019; Yacoub et al., 2018). In addition, uncertainty about the future was the main TD siblings' worry (Noonan et al., 2018; Paul et al., 2022; Corsano et al., 2017). Mixed results have been also found in quantitative studies: fulfilling sibling relationships and high engagement in shared activities have been revealed (Travers et al., 2020; Braconnier et al., 2018) in contrast to conflictual and low-quality sibling relationships (Hemati Alamdarloo et al., 2021).

As outlined recently in studies on adult TD siblings (Levante et al., 2025; Tomeny et al., 2017a; Eun Lee et al., 2019), sibling-focused parentification is the main determinant of the sibling relationship. Indeed, it has been reported that sibling-focused parentification, in the form of more caregiving duties and responsibilities assumed by TD siblings toward their brother/sister, is a potential predictor of low-quality sibling relationships (Tomeny et al., 2017b; Levante et al., 2023). Conversely, other studies on children and adult TD siblings (Eun Lee et al., 2019; Tomeny et al., 2017c, 2016) reported that the sibling relationship may benefit by caring for and taking responsibility for the TD sibling.

To date, no studies on TD siblings' reaction to the diagnosis process have been conducted despite the massive body of literature investigating this process in parents [e.g., (Lecciso et al., 2013b; McStay et al., 2014; Marvin and Pianta, 1996; Sher-Censor and Shahar-Lahav, 2022)]. According to Bowlby's (1982) Attachment Theory and Main and Hesse's (1990) Theory of Resolution of Loss and Trauma, Marvin and Pianta (1996) suggested that the resolution of a child's diagnosis of disability and/or chronic illness is essential for parents of children with a disability. The authors stated that parental reaction to the diagnosis process is shaped by their internal working models, consisting of the mental representations of themselves, their child, and the affective parent–child relationship. Typically, the parental reaction to the child's diagnosis process involves a sequence of emotional reactions beginning from shock and denial to contradictory emotions (e.g., anger, guilt, helplessness, and inadequacy) and the acceptance of the child's diagnosis (Kübler-Ross, 1970; Di Cagno et al., 1992). These reactions are the mirror of the parent's grief for losing the wished-for and healthy child (Siegel, 1996). It is worth noting that the acceptance of diagnosis is not reached by all parents. Indeed, the reaction to the diagnosis process has two outcomes: resolution *vs*. lack of resolution. In other words, resolved parents develop an accurate understanding of their child's strengths and difficulties, experience positive emotions, and have realistic hopes beyond the diagnosis while accepting their child for who he/she is. Conversely, unresolved parents struggle with grief, despair, confusion, and anger, often clinging to an idealized representation of the wished-for and healthy child. Parental physical and mental health [e.g., (Oppenheim et al., 2009; Reed and Osborne, 2018)], as well as the quality of the affective parent–child relationship (Oppenheim et al., 2009; Shah et al., 2011), was heavily affected by the reaction to the diagnosis process's outcome. In brief, the resolution of the diagnosis serves a protective role for parents and children, promoting the family's wellbeing and social adjustment, and encouraging responsiveness in the caregiving system (Sher-Censor and Shahar-Lahav, 2022). On the contrary, lack of resolution becomes a risk factor, as parents may exhibit prolonged grief and denial, which can lead to skewed perceptions of their child's abilities and needs (Marvin and Pianta, 1996; Sher-Censor and Shahar-Lahav, 2022; Oppenheim et al., 2009). Such dynamics can foster insecure or disorganized attachment patterns, further impacting the child's development and the broader family system (Biringen et al., 2014).

Considering the impact of the reaction to diagnosis on parents (Conway et al., 2017; John and Roblyer, 2017; Leblond et al., 2019) and the person with disabilities (Ahn et al., 2021; Kowalska et al., 2019; De Carlo et al., 2024; Martis et al., 2024), exploring this process in TD siblings not only opens the way to research investigations on the role of the reaction of diagnosis on the future caregiver of the individual with a disability (i.e., TD siblings) but also it is a pivotal public health issue in promoting targeted training. The lack of investigations in this field encourages the present study designed to probe whether TD siblings' resolution *vs*. lack of resolution of the brother/sister's diagnosis would predict the quality of affective sibling relationships in the form of higher closeness and lower levels of conflict, jealousy, self-marginalization, and worry. In addition, in line with the intergenerational transmission of internal working models (Bowlby, 1982), the study hypothesized that parental resolution *vs*. lack of resolution would be a potential predictor of the TD siblings' resolution *vs*. lack of resolution.

Due to the scarcity of studies on the role played by the sibship, the present study aimed at exploring whether being an older or younger TD sibling may affect the resolution and the quality of the affective sibling relationship.

The following sections review (a) the existing literature on parental reaction to the diagnosis process and its impact on the quality of the affective parent–child relationship in the context of disability and (b) the impact of the disability and socio-demographic factors on TD siblings' experience in terms of quality of affective sibling relationships in the context of disability.

### 1.1 The reaction to the diagnosis process on parents

During pregnancy, both parents created a mental representation of the phantasmatic child, which is related to the oedipal dimension of the parent's childhood experiences, and a mental representation of the imaginary child, which is shaped by the parents' current expectations (Lebovici, 1983; Ammaniti, 2008). Afterward, both these parental idealized child mental representations face the real child, and the consequent parental reaction affects the caregiver–child relationship (Lebovici, 1983; Ammaniti, 2008). The reactions and strategies applied by parents to face the real child are shaped by their internal working models (Bowlby, 1982), which are mental representations of themselves, their child, and the affective parent–child relationship. According to these mental representations, parents have expectations of parenthood and are responsive toward the child's needs.

In the context of the disability, the transition from the idealized child's mental representation to the real child with a disability requires special attention. There would be a child crystallization where the disability is perceived as an overwhelming hindrance, or the diagnosis may serve as a catalyst to address the disability-related challenge.

Based on Bowlby's (1982) Attachment Theory and Main and Hesse's (1990) Theory of Resolution of Loss and Trauma, the reaction to the child's diagnosis process provides a lens to understand this transition and examine the impact of disability on affective parent–child relationship and the whole family system. Four main emotional reactions are experienced by parents when the medical equipment communicates the child's diagnosis (Kübler-Ross, 1970; Di Cagno et al., 1992). The dynamic interplay of emotions, which could be different and conflicting among the parents, supports the non-linearity of this process. To begin with, shock and confusion are the initial emotional reactions: parents feel helpless and disrupted in their routines, and they believe that their child's life will be unhappy. The second emotional reaction is denial, which reflects the parents' refusal of the child's diagnosis, leading them to seek for further medical opinions, hoping a misdiagnosis. The third emotional reaction involves intense emotions such as anger, guilt, and shame, which can be flared out toward the medical equipment, the partner, or the child. The final reaction is the acceptance of the child's disability, which means that parents are aware of the child's strengths and difficulties, incorporate them into their pre-existing mental representations, and reorganize their lives accordingly. In addition, they set new goals for the whole family, focusing on the present and the future. This outcome of the reaction to the diagnosis process promotes a secure attachment bond, which, in turn, positively affects the quality of the affective parent–child relationship (Oppenheim et al., 2009; Shah et al., 2011).

Although this fourth reaction would be optimal for parents and children with disability's adjustment and wellbeing, assuming that it is reached by all parents is utopian. Indeed, some remain trapped in the earlier emotional reactions, exhibiting denial or anger. This lack of resolution about the child's disability may lead parents to deny or minimize his or her diagnosis, clinging to unrealistic expectations. As a result, these parents may seek alternative explanations for the child's disability, experience grief, despair, and confusion, and underestimate the heavy brunt of the diagnosis on themselves and their affective relationship with the child. Unresolved parents may misinterpret or downplay the child's needs and respond to them with withdrawal, overprotection, or intrusive behaviors (Marvin and Pianta, 1996). This reaction to the diagnosis process outcome promotes an insecure or disorganized attachment bond, which, in turn, negatively affects the quality of the affective parent–child relationship (Biringen et al., 2014).

To date, no studies have examined the role that parental resolution may have on other family members. Therefore, the present study focuses on the potential predictive role of parental resolution on that of the typically developing (TD) sibling.

### 1.2 Siblinghood in the context of disability

Traditionally, parents are the primary caregivers of the child with disabilities; nevertheless, during the lifespan, the experience of the TD siblings may be heavily affected by the disability. Evidence revealed a plethora of mixed effects. Heightened levels of anxiety, depression, social isolation, and behavioral disorders (Ross and Cuskelly, 2006; Giallo and Gavidia-Payne, 2006; O'Neill and Murray, 2016; Petalas et al., 2009; Rossetti and Hall, 2015; Sharpe and Rossiter, 2002; Stoneman, 2005; Dauz Williams et al., 2010) lead to low quality of life and wellbeing as well promoting the onset of internalizing and externalizing symptoms (O'Neill and Murray, 2016; Rossetti and Hall, 2015; Dauz Williams et al., 2010). Despite the TD siblings' caregiving burden related to the brother/sister's disabilities, positive outcomes have been identified: for instance, high levels of empathy, emotional sensitivity, psychosocial competence, and a sense of fulfillment because of the contribution provided to family management (Rossetti and Hall, 2015; Kaminsky and Dewey, 2001; Moyson and Roeyers, 2012; Opperman and Alant, 2003; Cuskelly and Gunn, 2003; Roper et al., 2014; Takataya et al., 2019; Pilowsky et al., 2004; Mulroy et al., 2008). Nevertheless, few studies (Cuskelly and Gunn, 2003; Hallion et al., 2018) found no significant differences between the experiences of TD siblings of people with and without disabilities.

Considering the key role played by the TD siblings in the present and future lives of people with disabilities, examining the potential factor(s) impacting the quality of sibling relationships is fundamental for research and clinical field (Travers et al., 2020; Braconnier et al., 2018; Levante et al., 2025; Tomeny et al., 2017a; Cuskelly, 2016; Floyd et al., 2016). Heterogeneous results have been achieved in the quality of the sibling relationship research. On the one side, there were close and supportive bonds fostered through shared recreational activities (Travers et al., 2020; Braconnier et al., 2018; Tomeny et al., 2017a; Cuskelly, 2016; Floyd et al., 2016). These bonds contribute to personal growth in terms of high levels of empathy, respect, and support (Bhattashali et al., 2018; Noonan et al., 2018; Paul et al., 2022; Stock et al., 2016; Tyerman et al., 2019; Yacoub et al., 2018). In addition, in adult TD siblings, self-esteem, family cohesion, and a sense of purpose (Paul et al., 2022) were promoted in navigating the challenges of a brother/sister's disabilities. On the other side, scholars highlighted experiences of conflict, rivalry, and emotional ambivalence, which can strain the quality of siblings (Avieli et al., 2019). In the context of disabilities, TD siblings may be worried about their and the brother/sister with disabilities' future, negatively encouraging TD siblings' social isolation (Noonan et al., 2018; Paul et al., 2022; Corsano et al., 2017) also due to the disability-related social stigma (Paul et al., 2022; Stock et al., 2016; Corsano et al., 2017). In brief, evidence brought to the fore both beneficial and detrimental effects of growing up with a brother/sister with disabilities on the TD sibling's experience and the quality of sibling relationships, highlighting the relevance of devoting attention to this vulnerable population.

Considering that the TD siblings' experience is the focus of the present study, the role played by several socio-demographic factors (i.e., gender, age, sibship, and the severity of the brother/sister's disability) is reviewed. According to previous studies on adolescents and adult TD siblings, gender comparisons showed that TD sisters reported both higher levels of anxiety and guilt (O'Neill and Murray, 2016; Yaldiz et al., 2021; Shivers and McGregor, 2019) and greater empathy, emotional involvement, self-efficacy, and overall wellbeing (Perenc and Peczkowski, 2018; Siman-Tov and Sharabi, 2023) than TD brothers. In contrast, a study on children and adolescents revealed that TD brothers tend to perceive sibling relationships as more conflictual than TD sisters do (Guidotti et al., 2021). On the TD siblings' age, research on children TD siblings showed that the caregiving demands related to the brother/sister disabilities placed during childhood lead to negative emotions and conflict in sibling relationships (Jones et al., 2006). In adolescence, shame and embarrassment were the main TD siblings' emotional reactions, which negatively promoted a low quality of affective sibling relationships (Corsano et al., 2017; Guidotti et al., 2021). Nevertheless, the evidence (Floyd et al., 2016; Petalas et al., 2015) outlined that in the transition into adulthood, the quality of the affective sibling relationship improved through greater satisfaction and a deeper appreciation of their caregiving experiences. Furthermore, despite the disability-related challenges, adult TD siblings reported a positive attitude toward their sibling relationship and the time spent with their brother/sister with disabilities (Eun Lee et al., 2019; Tomeny et al., 2017a). The study by Noonan et al. Noonan et al. (2018) reported interesting findings. The authors found that, despite their positive attitude, adults also reported significant distress about the future. This distress appeared to stem from the potential responsibility of acting as caregivers, particularly due to concerns about increased dependence on their brother/sister with a disability.

Early adulthood (i.e., 18–40 years) is the developmental stage selected in the present study, according to the lifespan theoretical model (Sugarman et al., 2003) because of its developmental tasks Havighurst (Havighurst, 1953): they are to make long-term decisions consisting of, for instance, choosing a career path, pursuing higher education, committing to a long-term relationship, deciding to start a family, achieving financial independence, or purchasing a home. In the context of disability, these developmental tasks may be particularly critical and hindered due to the TD siblings' caregiving duties and responsibilities toward the brother/sister with disabilities. Thus, a focus on this topic is needed.

A few studies investigated the impact of being a younger *vs*. an older TD sibling (i.e., sibship) on TD siblings' experience. Studies suggested that older TD siblings assumed more supportive roles than younger TD siblings (Hayden et al., 2023), labeling themselves as caregivers, mentors, and protectors (Nguyen et al., 2023) of the brother/sister with disabilities. In addition, younger TD siblings exhibited higher maladjustment in the form of anxiety and depressive symptoms than their older counterparts (O'Neill and Murray, 2016; Hastings, 2003).

Regarding the severity of the brother/sister disability, research (Knecht et al., 2015; Hallberg, 2013) reported that pervasive disability requiring more parental time and care increased loneliness in TD siblings which negatively affected the quality of the affective sibling relationship (Orsmond et al., 2009; Ross and Cuskelly, 2006). In other words, severe or profound disabilities lead parents to prioritize the child with the disability, and that, in turn, may determine an affective and emotional distance between parents and TD siblings because of the brother/sister's disability. Consequently, TD siblings may perceive themselves as not worthy of the parent's time and care and blow from family members.

## 2 The current study

The present study aims to preliminarily explore the TD siblings' experience in the context of the disability, testing two hypotheses (HP). First, based on the Marvin and Pianta (1996) theoretical framework demonstrating the relationship between parental resolution *vs*. lack of resolution and the quality of the affective parent–child relationship (Marvin and Pianta, 1996), we expected that the TD sibling's resolution *vs*. lack of resolution (resolution score) would be a potential predictive factor of the quality of the affective sibling relationship in terms of higher levels of closeness and lower levels of conflict, jealousy, self-marginalization, and worry.

Furthermore, considering that the reaction to the diagnosis process affects the parental internal working models (Marvin and Pianta, 1996) and that they are transmitted across generations (Bowlby, 1982), we expected that parental resolution score (i.e., resolution *vs*. lack of resolution) would be a potential predictive factor of the TD sibling's one.

In sum, the following HPs have been tested:

*HP1: The TD sibling's resolution score would be a potential predictive factor of the quality of the affective sibling relationship: the higher the resolution score, the higher the closeness and the lower the conflict, jealousy, self-marginalization, and worry*.

*HP2: The parental resolution score would be a potential predictive factor for the TD sibling's resolution score*.

**Figure 1:**
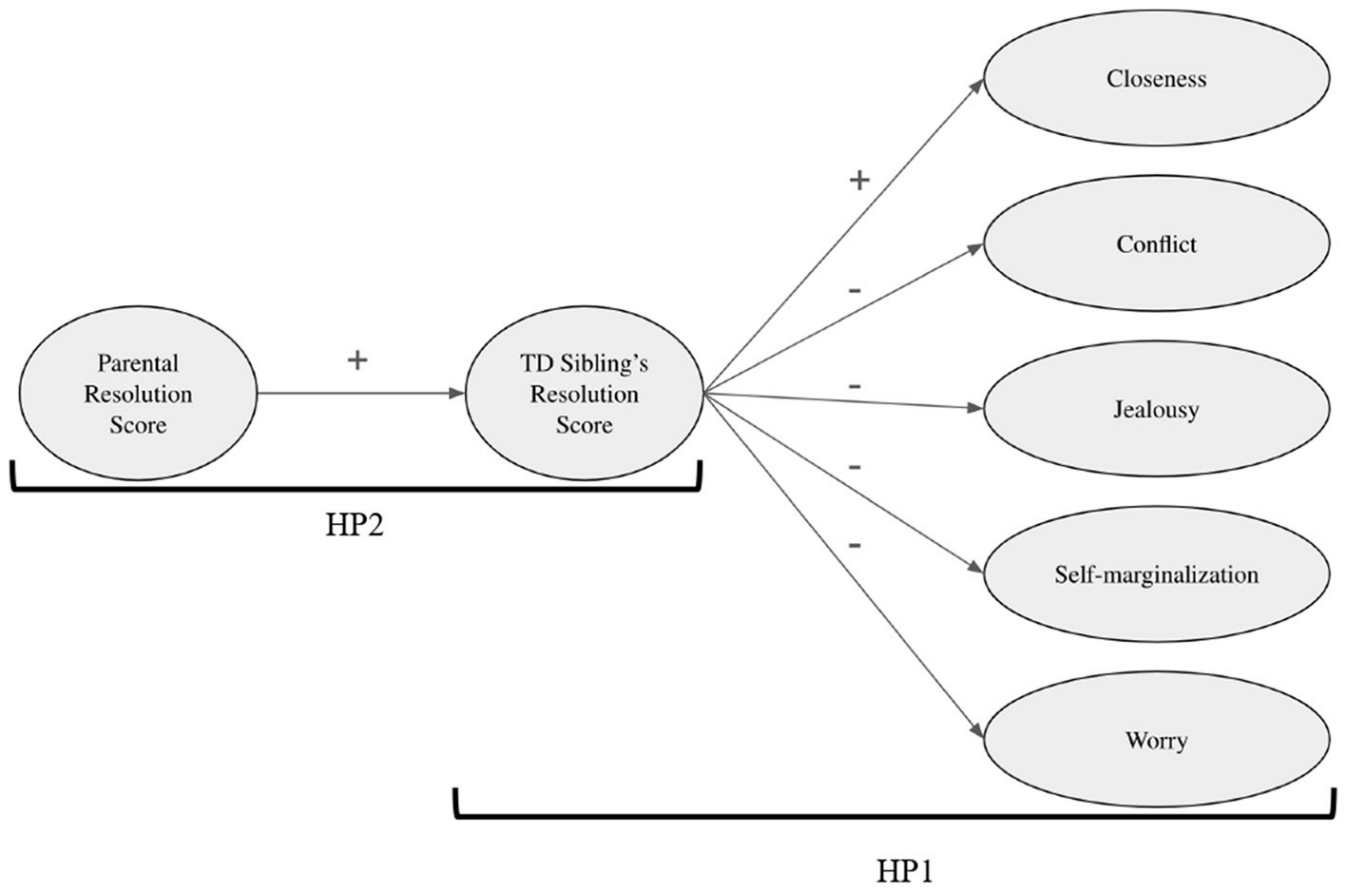
The hypothesized structural equation model.

Figure 1 shows the hypothesized model and the path direction (positive *vs*. negative).

Although the hypothesized model explored the role served by the main socio-demographic factors (i.e., gender, age, sibship, and the severity of the brother/sister's disabilities) on the TD siblings' experience (resolution score, quality of affective sibling relationship), the scarcity of research on the role served by the sibship requires further investigations. In this vein, the present study examined whether being the younger *vs*. older TD sibling may affect their resolution score and the quality of the affective sibling relationship. Two additional issues have to be considered in designing the present study: the type of brother/sister's disabilities and the TD siblings' age range involved in the present study. Neurodevelopmental disorders (NDDs, e.g., autism spectrum disorder, intellectual disability, and attention-deficit/hyperactivity disorder) and physical disability (e.g., blindness, multiple sclerosis, and motor disabilities) have been selected as macro groups explored in literature. Early adulthood (i.e., 18–40 years) is the developmental stage selected in the present study.

## 3 Materials and methods

### 3.1 Study design

Data were collected in Italy between April and September 2024 via an e-survey imported on LimeSurvey. Before the study, the University Ethical Committee for Research in Psychology at the Department of Human and Social Sciences of the University of Salento approved the research (No. 92949/2023). Participants signed an e-consent, and a spreadsheet informing them of their rights was provided. The target populations are parent–TD sibling dyads from families with a child with an NDD or a physical disability. A set of inclusion/exclusion criteria has been defined: parents have to (1) be over 18 years old, (2) have a son or daughter with an NDD or physical disability, and (3) have an 18-40-year-old child without a disability; TD siblings have to (1) be 18–40 years old, (2) have a brother/sister with an NDD or physical disability, and (3) not have any disabilities. Both parents and siblings have to be fluent in Italian language.

### 3.2 Statistical plan

No techniques for imputing missing data were computed due to the mandatory responses. The power analysis was calculated using the *jpower* package, the normality of data distribution was tested via the Shapiro–Wilk test, and the homogeneity of the variances was examined using Levene's test. Due to the non-Gaussianity of data, non-parametric tests (i.e., Mann–Whitney *U-tests*) were computed to investigate differences across gender, type of brother/sister's disability (disability group: TD siblings of people with NDDs *vs*. TD siblings of people with physical disability), and TD siblings' sibship (i.e., older TD sibling *vs*. younger TD sibling). Considering the unbalanced distribution of the TD siblings' developmental stage, no group comparison between early adulthood *vs*. adulthood was computed. Spearman's rho correlations among the dimensions were computed. The hypothesized model tests whether the TD siblings' resolution score would predict the quality of affective sibling relationship (HP1) and whether the parental resolution score would predict the TD siblings' resolution score (HP2). The TD siblings' sibship (RQ), gender, age, and the severity of the brother/sister's disorder/disability were included as control variables. The following goodness-of-fit indices were used to evaluate the structural equation model: the chi-square-value (**χ**^2^), the Comparative Fit Index (CFI), and the root mean square error of approximation (RMSEA). Given that the χ2 value is influenced by the sample size, it was considered, together with CFI and RMSEA. Byrne (2016) suggested accepting a model when the CFI is higher than 0.90 and close to 0.95 and when the RMSEA is 0.08 or less. Regarding the effect size of beta coefficients in the structural equation model, effect sizes between 0.10 and 0.29 are said to be only small, effect sizes between 0.30 and 0.49 are medium, and effect sizes of 0.50 or greater are large (Cohen, 1988; Fey et al., 2023). Statistical analyses were computed using Jamovi. (2024).

### 3.3 Participants

Three hundred and sixty-five parent–sibling dyads completed an e-survey [parents: *M*_*age*_(*SD*) = 51.2 (6.95) years, age range = 25–64 years; mothers = 78.4%; TD siblings: *M*_*age*_ (*SD*) = 23.2 (3.60) years, age range = 18–39 years: to be accurate, early adults *n* = 289, adults *n* = 76; females = 53.7%]. One hundred and seventy parent–sibling dyads were from families of people with an NDD [*M*_*age*_ (*SD*) = 20.3 (6.67) years, age range = 4–50 years; females = 11.5%], and one hundred and ninety-five parent–sibling dyads were from families of people with a physical disability [*M*__*ag*_e_ (*SD*)= 22.9 (6.15) years, age range = 2–42 years; females = 24.9%].

In families of individuals with NDD, among TD siblings, 38.82% are employed; of them, 3.03% are with a low educational level, 57.58% with an intermediate level, and 39.39% with a high educational level), whereas 61.18% of TD siblings of individuals with NDD are not employed: of them, 15.38% with a low educational level and 84.62% with an intermediate educational level. Among parents, 62.94% are employed: of them, 15.89% with a low educational level, 61.68% with an intermediate level, and 22.43% with a high level of education, whereas 37.06% of parents are not employed: of them, 41.27% with a low educational level and 58.73% with an intermediate level. Finally, among individuals with NDD, 10.59% are employed: of them, 5.56% with a low educational level and 94.44% with an intermediate level, whereas 89.41% are not employed: 13.16% without a degree, 39.47% with a low educational level, 46.05% with an intermediate level, and 0.66% with a high educational level.

In families of individuals with physical disability, among TD siblings, 47.69% are employed: of them, 3.23% with a low educational level, 64.52% with an intermediate level, and 32.26% with a high educational level, whereas 52.31% of TD siblings are not employed: 6.86% with a low educational level, 81.37% with an intermediate educational level, and 11.76% with a high educational level. Among parents, 62.56% are employed: of them, 23.77% with a low educational level, 50.82% with an intermediate level, and 25.41% with a high level of education, whereas 37.44% of parents of individuals with physical disability are not employed: of them, 43.84% with a low educational level, 52.05% with an intermediate level, and 4.11% with a high educational level. Finally, among individuals with a physical disability, 17.95% are employed: 5.71% with a low educational level, 77.14% with an intermediate level, and 17.14% with a high level of education, whereas 82.05% are not employed: of them, 8.13% without a degree, 27.50% with a low educational level, 58.13% with an intermediate level, and 6.25% with a high educational level.

Table 1 provides a summary of socio-demographic details.

**Table 1:** Socio-demographic information.

**Educational level**	
Parents	Low (up to 8 years of education) = 24.49% Intermediate (up to 13 years of education) = 55.62% High (13 or more years of education) = 15.89%
TD siblings	Low (up to 8 years of education) = 6.85% Intermediate (up to 13 years of education) = 70.14% High (13 or more years of education) = 36.01%
Individuals with NDD	No formal education = 11.76% Low (up to 8 years of education) = 35.88% Intermediate (up to 13 years of education) = 51.76% High (13 or more years of education) = 0.59%
Individuals with physical disabilities	No formal education = 6.67% Low (up to 8 years of education) = 23.59% Intermediate (up to 13 years of education) = 61.54% High (13 or more years of education) = 8.21%
**Marital status**	
Parents	With a partner = 85.91% Without a partner = 14.79%
TD siblings	With a partner = 13.15% Without a partner = 86.85%
Individuals with NDD	With a partner = 5.29% Without a partner = 94.71%
Individuals with physical disabilities	With a partner = 7.69% Without a partner = 92.31%
**Employment status**	
Parents	Employed = 62.74% Unemployed = 37.26%
TD siblings	Employed = 43.56% Unemployed = 56.44%
Individuals with NDD	Employed = 10.59% Unemployed = 89.41%
Individuals with physical disabilities	Employed = 17.95% Unemployed = 82.05%
**Sibship**	
Older *vs*. younger TD sibling	Older TD sibling = 59.2% Younger TD sibling = 40.8%

### 3.4 Measures

#### 3.4.1 Parental measures

##### 3.4.1.1 Parental resolution score

The 42-item Reaction to Diagnosis Questionnaire (Sher-Censor et al., 2020) is a self-report questionnaire developed to assess parents' resolution of their child's diagnosis. For the present study, the original version of the questionnaire was provided by the developers via personal communication, and a back translation in the Italian language of the questionnaire was made. Following Marvin and Pianta's theoretical framework, the items reflect indicators of resolution (e.g., “*Today I can see my child's difficulties as well as his/her strengths and achievements*” and “*I feel that my feelings regarding my child's diagnosis have changed since my child received the diagnosis*”) and lack of resolution (e.g., “I am angry about everything that happened to my child and me” and “*It is difficult for me to stop thinking about my child's diagnosis and difficulties*”). The response was rated on a 5-point Likert scale (1 = strongly disagree; 5 = strongly agree). Good internal consistency across two different disabilities (Sher-Censor et al., 2020) was demonstrated. The resolution score is calculated as the average of all items, with a higher score indicating a higher degree of resolution of the diagnosis (*M*_*age*_ = 3.78; *SD* =0.43; α =0.89).

#### 3.4.2 Typically developing sibling measures

##### 3.4.2.1 TD sibling's resolution score

The parental Reaction Diagnosis Questionnaire was adapted to assess the TD siblings' resolution score for the present study's purposes. The Italian adaptation was carried out by replacing references to one's child with references to one's brother/sister with disabilities: for instance, the item was adapted from “*In spite of the difficulties, I see that my child is successful in facing his/her challenges*” to “*In spite of the difficulties, I see that my brother/sister with a disability is successful in facing his/her challenges*.” Similar to the questionnaire for parents, this adapted measure assesses the TD siblings' resolution score about the brother/sister's diagnosis in the forms of indicators of resolution (e.g., “*I feel that my brother/sister's condition is improving*” or “*I shared my brother/sister's diagnosis with my extended family*”) and lack of resolution (e.g., “*I don't believe that my brother/sister's level of independence will improve in the future*” or “*I am angry about everything that happened to my brother/sister and me*”). The response rate varied on a 5-point Likert scale (1 = strongly disagree; 5 = strongly agree). The resolution score is calculated as the average of all items, with higher scores indicating a higher degree of resolution of the diagnosis [*M*_*age*_ = 3.89, *SD* = 0.41; α = 0.89].

##### 3.4.2.2 Quality of affective sibling relationship

The 23-item Siblings' Experience Quality Scale (Sommantico et al., 2020) (the questionnaire was provided by the developers via personal communication) is a self-report questionnaire designed to assess TD siblings' emotional, behavioral, and cognitive experiences related to the sibling relationship in the disability context. The Siblings' Experience Quality Scale provided five subscales: (1) Closeness (*n* = 5 items; e.g., “*I tell my sister/brother that she/he is important to me*”), (2) Conflict (*n* = 5 items; e.g., “*I am often bothered by my brother's or sister's behavior*”), (3) Jealousy (*n* = 5 items; e.g., “*I have often been jealous of the way my parents have treated my brother/sister*”), (4) Self-Marginalization (*n* = 3 items; e.g., “*I often feel that I don't have to give worries to my parents*”), and (5) Worry (*n* = 5 items; e.g., “*I think my brother/sister's emotional life outside the family will not be easy*”). Responses options are rated on a 5-point Likert scale (1 = strongly disagree; 5 = strongly agree). Each subscale is calculated by averaging the items, with higher scores reflecting greater levels of Closeness [*M*_*age*_ = 6.26, *SD* = 0.77; α = 0.75], Conflict [*M*_*age*_= 2.44, *SD* = 1.26; α = 0.88], Jealousy [*M*_*age*_ = 2.30, *SD* = 1.19; α = 0.80], Self-Marginalization [*M*_*age*_ = 4.67, *SD* = 1.44; α = 0.77], and Worry [*M*_*age*_ = 3.19, *SD* = 1.56; α = 0.88].

##### 3.4.2.3 Severity of disability

The 6-item Barthel Index (Mahoney and Barthel, 1965) assesses the severity of disability based on needs in activities of daily living (e.g., eating, dressing, and personal hygiene care). Responses options are rated on a 5-point Likert scale (1 = completely independent; 5 = unable to act). The total score is calculated as the sum of all items, with higher scores indicating greater severity of disability [*M*_*age*_ = 3.57, *SD* = 1.23; α = 0.94].

## 4 Results

### 4.1 Preliminary analyses

The power analysis revealed that the sample size (*n* = 365) is adequate for drawing valid conclusions regarding significant effects, ensuring the robustness and the interpretations of results (δ ≥ 0.5 with a probability of at least 0.9). Shapiro–Wilk test for parental and sibling resolution scores and quality of relationship dimensions was significant. Levene's test for homogeneity is non-significant; thus, variances are roughly equal, and the assumption of homogeneity is tenable. Group comparisons are tabulated (Table 2). Gender differences have been found only for closeness and self-marginalization subscales of quality of sibling relationship: to be accurate, TD sisters reported greater closeness and self-marginalization than brothers. No disability-group (NDDs *vs*. physical disability) differences have been revealed. On sibship, the results showed that younger TD siblings reported a higher level of sibling resolution score than the older ones.

**Table 2:** Mann–Whitney *U*-tests.

**Psychological variables**	**TD sibling**			**Disability group**			**Sibship**		
	**Sisters M(SD)**	**Brothers M(SD)**	***U; p***	**NDD group M(SD)**	**Physical Group M(SD)**	***U; p***	**Older M(SD)**	**Younger M(SD)**	***U; p***
TD sibling's resolution score	3.92 (0.39)	3.86 (0.43)	1.541	3.90 (0.40)	3.88 (0.42)	1.603	3.84 (0.43)	3.96 (0.38)	1.357^**^
Parental resolution score	3.81 (0.41)	3.75 (0.46)	1.550	3.77 (0.43)	3.80 (0.44)	1.582	3.76 (0.44)	3.81 (0.42)	1.556
Closeness	6.33 (0.75)	6.17 (0.78)	1.431^*^	6.24 (0.80)	6.26 (0.75)	1.620	6.19 (0.81)	6.36 (0.70)	1.396
Conflict	2.45 (1.30)	2.44 (1.20)	1.634	2.50 (1.26)	2.39 (1.24)	1.567	2.41 (1.24)	2.49 (1.27)	1.552
Jealousy	2.35 (1.27)	2.24 (1.09)	1.613	2.34 (1.16)	2.26 (1.21)	1.566	2.27 (1.15)	2.33 (1.23)	1.591
Self-marginalization	4.89 (1.34)	4.40 (1.51)	1.337^***^	4.79 (1.42)	4.56 (1.45)	1.513	4.70 (1.38)	4.62 (1.52)	1.570
Worry	3.20 (1.54)	3.17 (1.57)	1.627	3.28 (1.54)	3.11 (1.56)	1.559	3.30 (1.55)	3.02 (1.55)	1.442

^*^p < 0.05, ^**^p < 0.01, and ^***^p < 0.001.

### 4.2 Correlations between parental resolution score, sibling resolution score, and quality of affective sibling relationship

Table 3 reported correlations between variables. The results showed that the parental resolution score was positively associated with the TD siblings' resolution score; in other words, the higher the parental resolution score, the higher the TD siblings' resolution score. In addition, a positive association was reached between TD siblings' resolution score and closeness in sibling relationships, whereas a negative correlation between TD siblings and conflict, jealousy, self-marginalization, and worry in sibling relationships has been revealed. This means that the higher the TD siblings' resolution scores, the higher closeness and lower conflict, jealousy, self-marginalization, and worry.

**Table 3:** Correlation between study variables.

**Psychological variables**	**(1)**	**(2)**	**(3)**	**(4)**	**(5)**	**(6)**	**(7)**	**(8)**	**(9)**	**(10)**
TD sibling's resolution score	0.687^***^	0.280^***^	−0.222^***^	−0.367^***^	−0.275^***^	−0.540^***^	−0.114^*^	−0.350^***^	−0.133^**^	0.060
Parental resolution score (1)	–	0.226^***^	−0.221^***^	−0.348^***^	−0.248^***^	−0.526^***^	−0.038	−0.323^***^	−0.028	0.055
Closeness (2)		–	−0.354^***^	−0.379^***^	−0.021	−0.195^***^	−0.045	−0.013	−0.114^*^	0.118^*^
Conflict (3)			–	0.539^***^	0.129^***^	0.149^**^	−0.113^*^	−0.137^**^	−0.030	−0.012
Jealousy (4)				–	0.285^***^	0.400^***^	−0.011	0.044	−0.009	0.023
Self-marginalization (5)					–	0.364^***^	−0.030	0.082	0.021	0.167^**^
Worry (6)						–	0.055	0.538^***^	0.088	0.016
TD sibling's age (7)							–	0.097	0.244^***^	−0.194^***^
Severity of disability of the brother/sister (8)								–	−0.45	−0.102
Sibship (9)									–	−0.033
TD sibling gender (10)										–

^*^p < 0.05, ^**^p < 0.01, and ^***^p < 0.001.

TD sibling: typically developing sibling. Sibship: The older TD sibling was coded with 1, younger TD sibling was coded with 0. TD sibling gender: TD sister was coded with 1, and TD brother was coded with 0.

### 4.3 Parental resolution score, TD sibling's resolution score, and their impact on quality of affective sibling relationship: structural equation model results

Due to the lack of difference between the TD siblings of a brother/sister with NDDs and physical disability in all study's variables, the two hypotheses and the research question were tested on the whole sample. The results showed good fit indices (χ^2^ = 24.9; *df* = 5; *p* < 0.001; *CFI* = 0.97; *RMSEA* = 0.10). Beta coefficients, 95% confidence intervals, and standard errors were tabulated (Table 4).

**Table 4:** Betas coefficients, 95% confidence intervals, and standard errors of the structural equation model.

**Path**		***B*; [95% CI]**	**SE**
**(HP2)** Parental resolution score → TD sibling's resolution score		0.648^***^ [0.588; 0.707]	0.036
**(HP1)** TD sibling's resolution score →	Closeness	0.29^***^ [0.196; 0.396]	0.098
	Conflict	−0.283^***^ [−0.382; −0.188]	0.159
	Jealousy	−0.414^***^ [−0.506; −0.321]	0.146
	Self-marginalization	−0.33^***^ [−0.427; −0.234]	0.180
	Worry	−0.445^***^ [−0.523; −0.357]	0.161
**Control variables**→**study variables**			
TD sibling gender →	TD sibling's resolution score	0.001 [−0.073; 0.076]	0.031
	Closeness	0.094 [−0.005; 0.195]	0.080
	Conflict	−0.025 [−0.126; 0.074]	0.129
	Jealousy	0.052 [−0.045; −0.150]	−119
	**Self-marginalization**	0.187^***^ [0.089; 0.284]	0.146
	Worry	0.073 [−0.008; 0.155]	−130
TD sibling age →	TD sibling's resolution score	−0.072 [−0.149; 0.003]	0.004
	Closeness	0.054 [−0.049; 0.158]	0.011
	**Conflict**	−0.11^*^ [−0.213; −0.007]	0.018
	Jealousy	−0.050 [−0.151; 0.049]	0.016
	Self-marginalization	−0.032 [−0.134; 0.069]	0.020
	Worry	−0.018 [−0.102; 0.066]	0.018
Severity of brother/sister disability →	**TD Sibling's Resolution Score**	−0.128^***^ [−0.203; −0.053]	0.013
	**Closeness**	0.112^*^ [0.010; 0.214]	0.033
	**Conflict**	−0.238^***^ [−0.338; −0.138]	0.054
	**Jealousy**	−0.120^**^ [−0.219; −0.021]	0.050
	Self-marginalization	−0.019 [−0.120; 0.080]	0.061
	**Worry**	0.340^***^ [.260; 0.421]	−0.54
**(RQ)** TD sibling sibship →	**TD sibling's resolution score**	−0.090^*^ [−0.164; −0.016]	0.031
	Closeness	−0.073 [−0.173; 0.027]	0.080
	Conflict	−0.055 [−0.155; 0.044]	0.130
	Jealousy	−0.073 [−0.170; 0.024]	0.120
	Self-marginalization	−0.008 [−0.107; 0.090]	0.147
	Worry	0.041 [−0.040; 0.123]	0.132

^*^p < 0.05, ^**^p < 0.01, ^***^p < 0.001.

Overall, the parental resolution has a large effect on TD siblings' resolution scores. This means that the parental resolution may be a potential positive predictor of the resolution of the diagnosis of the brother/sister with a disability on behalf of the TD siblings.

Medium-small effects have been revealed between the TD siblings' resolution score and the subscales of the quality of the sibling relationship. Albeit preliminary, the TD siblings' resolution may be a potential protective factor for a high-quality sibling relationship. In other words, resolved TD siblings perceived not only a close sibling relationship but also low conflict, jealousy, self-marginalization, and worry. It is worth noting that among them, jealousy and worry are the subscales of the quality of the sibling relationship that benefit most from the TD siblings' resolution: the higher the TD resolution, the lower the jealousy and worries.

Concerning the role of the control variables, medium-small effects have been outlined. To begin, on the TD siblings' resolution, only the sibship served a role: the older TD siblings reported a low degree of resolution of the diagnosis. Nevertheless, the effect size is small. On conflict and self-marginalization subscales of the sibling relationship, TD siblings' age and gender have a small effect, respectively. In other words, older TD siblings experienced low conflict in sibling relationships and TD sisters perceived high levels of self-marginalization. Finally, the medium-small effect outlined when the impact of the severity of the disability was considered: TD siblings of a brother/sister with a profound (NDD or physical) disability reported a low degree of resolution of the diagnosis, a close but also worried sibling relationship; nevertheless, no conflict and jealousy in sibling relationships were retrieved. It is worth noting that worry is the subscale of the quality of the sibling relationship that benefits most from the TD siblings' resolution.

In sum, despite the explorative nature of the study, the results are promising and support the study's HPs and RQ.

## 5 Discussion

In the context of disability, according to the systemic approach (Minuchin, 1985), it may be pivotal for public health issues to investigate the reaction to the diagnosis process not only parents (Sher-Censor and Shahar-Lahav, 2022; Conway et al., 2017; John and Roblyer, 2017; Leblond et al., 2019; Lecciso et al., 2013a or the persons with a disability (Ahn et al., 2021; Kowalska et al., 2019; De Carlo et al., 2024; Martis et al., 2024) but also on the TD siblings who will be the future caregiver of the persons with a disability. No studies have been conducted on this topic. Thus, the present study aims to explore this novel research field by investigating two novel paths of relationships. To begin with, the potential protective role served by the TD siblings' resolution score on the quality of the affective sibling relationship has been explored (HP1); in addition, the likelihood of transmitting the adapted internal working models resulting from the parental resolution of the diagnosis to TD siblings has been investigated (HP2); a further cutting-edge issue was examining the role of the sibship (being an older *vs*. a younger TD sibling) in TD siblings' resolution score and quality of the affective sibling relationship (RQ).

Preliminary results indicated no differences in parental and TD siblings' resolution scores and the quality of sibling relationships between the two disability groups (i.e., NDD *vs*. physical disabilities). Considering the explorative nature and the novelty of the current study, the results warrant further investigations. However, previous research (Lecciso et al., 2013b; Marvin and Pianta, 1996; Sher-Censor and Shahar-Lahav, 2022; Poslawsky et al., 2014; Pianta et al., 1999; Hutman et al., 2009) on parents suggested that resolution scores are primarily influenced by the severity of the child's disability rather than the type (e.g., NDD, physical disability, and chronic illness). Similar to parents, this pattern may also be extended to TD siblings. In addition, previous research (Orsmond et al., 2009; Ross and Cuskelly, 2006) devoting attention to the TD siblings' experiences, revealed that the severity of the child's disorder/disability affected the quality of sibling relationships more than the type of disability. It is worth noting that the lack of study exploring the role of the resolution in TD siblings did not allow for discussion of the results in light of existing literature.

Nevertheless, further investigations are needed. In addition, the results emphasized that TD sisters perceived a closer sibling relationship and experienced more self-marginalization than their male counterparts. This coin has two sides. On the one hand, the result may highlight the benefit of the caregiving role served by the TD sisters toward the brother/sister with disabilities: although the disability-related responsibilities and duties, the time spent together may enhance closeness in sibling relationships. Nevertheless, it may be worth noting that the TD sisters' self-marginalization may reflect their distress, which may be due to the awareness that they cannot mess and/or they must not be a worry for overwhelmed parents. Conversely to other studies recruiting mainly TD sisters (Travers et al., 2020; Braconnier et al., 2018; Levante et al., 2023; Chiu, 2022), our result has been reached in a gender-balanced sample supporting this potential 2-fold interpretation and opening the way to additional investigations on the topic. Younger TD siblings were more resolved than older ones. Growing up with an older brother/sister with a disability may increase the attitude toward disability on behalf of the younger TD siblings, perceiving it as a part of the familiar context and their own life. No empirical studies have been carried out on this topic; thus, further investigations are required to deepen the determinants engaged in this relationship.

Due to the lack of difference across disability groups (NDD *vs*. physical disability) on all considered variables, the model has been tested on the whole sample. Correlational coefficients and the tested model supported both hypotheses. Regarding the HP1, the results revealed that the TD sibling's resolution score was a potential protective factor for the quality of sibling relationships characterized by high closeness and low levels of conflict, jealousy, self-marginalization, and worry. Similar to resolved parents [e.g., (Sher-Censor and Shahar-Lahav, 2022; Oppenheim et al., 2009; Reed and Osborne, 2018; Sher-Censor and Shahar-Lahav, 2022)], resolved TD siblings may perceive their brother/sister with disabilities' strengths and difficulties and, in turn, may be more aware of what they could expect from him or her in the sibling relationship. The TD siblings' resolution of the brother/sister's disability may lead to adapting the mental representations of the sibling relationship, which may be characterized by closeness despite the bothered but unwitting behaviors of the brother/sister with a disability. In addition, the resolved TD siblings may be aware of the brother/sister with disability's difficulties and consequently understand the reasons for the parental lack of attention, leaving behind jealousy toward the brother/sister with a disability. The results also highlighted that the TD siblings' resolution might be a potential protective factor for TD siblings' self-marginalization and worry. This means that TD siblings may be less withdrawn and perceive themselves as fallible human beings who need and receive parental support. Furthermore, the resolved TD siblings may not be discouraged about the future of their brother/sister with a disability but consider the positive side despite the brother/sister's difficulties.

Nevertheless, the potential protective role served by the TD siblings' resolution score on the sibling relationship may open the way for consideration of the quality of the parent–TD sibling relationship. Despite the disability-related challenges, parents are required to face a 2-fold task toward the TD siblings, that is, being their secure base and communicating overtly on the brother/sister with a disability strength and difficulty. Although they may be harsh parental tasks, parents may support TD sibling's resolution. This consideration is closely related to the study's hypothesis (HP2): indeed, the potential predictive role of the parental resolution score on the TD sibling's resolution has been investigated. The results supported the second hypothesis (HP2), suggesting that the parental adapted internal working model resulting from the resolution of the child's diagnosis may be transmitted to TD siblings. Therefore, following parents in reaction to the diagnosis process when the diagnosis is communicated by professionals may have considerable cascade effects on each family member. Parents may transmit their adapted mental representation of the child with a disability, helping the TD sibling to adapt their own related to a healthy brother/sister. Thus, being a secure base and resolved parents able to have out not only on challenges but also on the benefits of growing up with a brother/sister with a disability is mandatory. While these two novel paths of relationship provide promising and encouraging results, additional studies are needed.

A further novel issue addressed by the present study regards the role served by the sibship (being the older TD sibling *vs*. the younger TD sibling) on the TD sibling's resolution and the quality of the sibling relationship. The results supported the group comparison, revealing that the younger TD siblings reported high-resolution scores. Hence, growing up with a brother/sister with disabilities as a younger TD sibling may be a potential promoting factor for the resolution of the diagnosis. A reflection arises from this result: culturally, there is the expectation that the older sibling would be those who will care for vulnerable family members, e.g., elder parents and/or brother/sister with disabilities (Nuckolls, 1993; Weisner et al., 1977; Zukow-Goldring, 1995). Thus, the result reached in the present study may suggest that the older TD siblings may deem as mandatory the caregiver demands; whereas the younger TD siblings who may experience the disability as a part of their life may face them more spontaneously, resolving the diagnosis. It is worth noting that this consideration has to be read carefully due to the fact this is the first study exploring the role of these socio-demographic factors on the unexplored construct of the TD resolution.

Further considerations are related to the impact of the TD sibling's gender and age, and the severity of the disability of the brother/sister on the TD sibling's resolution score and quality of affective sibling relationship. In sum, TD sister reported higher levels of self-marginalization compared to the TD brothers: the certainty that parents do not have time and/or they are not available to support them because the disability-related challenges and duties may lead the TD sisters to withdraw, to not call for help, and/or to overburden of each responsibility. The sequelae on the TD sister's experience may be spotless. It is worth noting that, conversely to other studies recruiting mainly TD sisters [e.g., (Travers et al., 2020; Levante et al., 2025; Chiu, 2022)], the gender-balance sample in the present study may corroborate the traditional care role served by TD sisters in caring [e.g., (Greenberg et al., 1999; Hodapp et al., 2010; Orsmond and Seltzer, 2000)] and their vulnerability to mental health as well (O'Neill and Murray, 2016; Yaldiz et al., 2021; Shivers and McGregor, 2019). Indeed, following the Social Role Theory (Eagly and Koenig, 2009; Pinho and Gaunt, 2024), societal expectations and norms lead females to internalize the role of primary family caregivers, responsible for managing household duties and attending to the needs of family members. In light of these results, it is crucial to further explore the gendered dynamics of caregiving roles, particularly the psychological impact on TD sisters, to better understand how these experiences shape their wellbeing and the family system.

Concerning the TD siblings' age, our results suggested that older TD siblings reported lower levels of conflict in the sibling relationship compared to younger counterparts. This aligns with previous research on the quality of sibling relationships across the lifespan (Corsano et al., 2017; Guidotti et al., 2021; Jones et al., 2006; Chiu, 2022), which highlighted that older TD sibling who reported awareness of parents' caregiver burden of a child with a disability were more aware of brother/sister's disability-related challenges mitigating the conflicts in the sibling relationship.

Regarding the severity of the disability, the results outlined two opposing viewpoints: on the one hand, the greater severity of the brother/sister's disability may be a risk factor for the TD siblings' resolution. Similar to parents (Lecciso et al., 2013b; Poslawsky et al., 2014; Rentinck et al., 2010; Schuengel et al., 2009; Yaari et al., 2017), the more profound the disability, the lower the TD sibling's resolution. For example, a profound disability with severe impairments in the main developmental domains may prevent or hide the awareness of the brother/sister with a disability's strengths. As a consequence, the TD siblings may focus mainly on the brother/sister's difficulties which, in turn, may be experienced as insurmountable hurdles. On the other hand, the greater severity was associated with increased closeness and reduced jealousy despite the worries in the sibling relationship. Hence, the caregiving demands associated with having a brother/sister with disabilities contribute not only to a sense of burden but also strengthen solidarity and emotional connection in sibling relationships.

## 6 Strengths, limitations, and future directions

While further investigations are needed, the foremost strengths of the study regard the preliminary investigation of the unexplored reaction to the diagnosis process in the TD sibling population: their potential impact on the sibling relationship. In addition, the potential predictive role of the parental resolution to the TD siblings' resolution emerges as an important factor worthy of further consideration. Considering the systemic approach (Minuchin, 1985), these preliminary results may support the need to understand family dyadic relationships comprehensively.

The results of this study should be interpreted in light of limitations. The parent sample predominantly consists of mothers. Future research should aim to include a more balanced sample of parents or focus specifically on fathers. The study did not include a comparison group of parent–sibling dyads from families with typically developing individuals. The cross-sectional design limits the generalizability of the results. In addition, the use of convenience sampling introduces a potential selection bias. The severity of the disability was assessed based on the individual's needs in activities of daily living. Future research should also consider the degree of cognitive and/or linguistic impairment. This study focused on early adulthood and adulthood, but sub-groups analyses were not computed; future research should incorporate a more detailed stratification within this age range. Finally, this study did not collect specific information regarding the type of diagnosis within the NDD or physical disability categories. Future studies should further investigate the resolution of the diagnosis from the perspective of TD siblings, considering specific diagnoses.

The results open the way for future directions. Interventions could be designed to promote the TD siblings' resolution of the diagnosis, for instance, promoting their adaptability of the mental representations promoting theory of mind skills (Marchetti et al., 2014; Petrocchi et al., 2021) which could improve the perception of oneself, the brother/sister with a disability, and family relationships, fostering greater emotional awareness and enhancing interpersonal dynamics within the family. Due to the predictive role of the parental resolution on the TD sibling's resolution, a future field of research could investigate whether the parental resolution could be a promoting factor for persons with disabilities resolution. Furthermore, this study administered measures grounded in Bowlby's attachment theory (Bowlby, 1982) and Marvin and Pianta's frameworks (Marvin and Pianta, 1996). It would be interesting to deepen their applicability in psychodynamic and systemic approaches, both in research and clinical practice.

Finally, considering the TD sibling's vulnerability to psychopathology (Levy-Wasser and Katz, 2004), future studies could investigate the impact of the resolution of the diagnosis on their mental health in terms of wellbeing and emotional and behavioral adjustment.

## Data Availability

The raw data supporting the conclusions of this article will be made available by the authors, without undue reservation.
